# Argumentium Ad Hominem

**Published:** 1875-08

**Authors:** 


### ARGUMENTUM AD HOMINEM.

"What ails papa's mouf?" said a sweet little girl,
Her bright eyes revealing her teeth white as pearl;
"I love him, and kiss him, and sit on his knee,
But the kisses don't smell good, as kisses should be.

"But mamma"—her eyes opened wide as she spoke—
"Do you like nasty kisses of 'bacco and smoke?
They might do for boys, but for ladies and girls
I dont think them nice," said she shaking her curls.

"Don't nobody's papa have moufs nice and clean?
With kisses like yours, mamma, that's what I mean;
I want to kiss papa, I love him so well,
But kisses don't taste good that have such a smell.

"It's nasty to smoke, and eat 'bacco, and spit,
And the kisses aint good, and aint sweet, not a bit!"
And her blossom-like face wore a look of disgust,
As she gave out her verdict so earnest and just.

Yes, yes, little darling! your wisdom has seen
That kisses for daughter and wife should be clean;
For kisses lose something of nectar and bliss
From mouths that are stained and unfit for a kiss.

—*Herald of Health.*

